# Variations of venous drainage of the thyroid gland and their surgical implications: a narrative review

**DOI:** 10.1590/1677-5449.202201632

**Published:** 2023-04-11

**Authors:** Rajani Singh

**Affiliations:** 1 Uttar Pradesh University of Medical Sciences, Department of Anatomy, Etawah, Uttar Pradesh, India.

**Keywords:** anatomy, thyroid gland, thyroid veins, venous drainage, anterolateral neck, anatomia, glândula tireoide, veias tireóideas, drenagem venosa, pescoço anterolateral

## Abstract

Diseases of the thyroid are common worldwide, so knowledge of its normal and variant anatomy, especially of the veins of thyroid, is essential for safe and successful surgery involving the anterolateral neck. The aim of this study is to consolidate all information related to venous drainage of the thyroid gland as a ready reference for vascular and endocrine surgeons. The study was conducted at the Department of Anatomy and the literature search was carried out using the Pubmed, Scielo, Researchgate, Medline, and Scopus databases. Various terms related to the thyroid gland and its venous drainage were used to explore the literature. The literature review revealed that the superior and middle thyroid veins have the fewest variations in terms of course and termination while the inferior thyroid vein has the most variations in terms of course and termination. Detailed knowledge of normal and variant anatomy of the thyroid veins is of utmost use for vascular surgeons performing anterolateral neck surgery, especially tracheostomy, a lifesaving procedure, minimizing intraoperative and postoperative complications and morbidity and mortality.

## Resumo

As doenças da tireoide são comuns em todo o mundo; portanto, o conhecimento tanto da anatomia normal quanto de variações anatômicas, especialmente das veias tireóideas, é essencial para uma cirurgia segura e bem-sucedida da região anterolateral do pescoço. O objetivo deste estudo é consolidar todas as informações relacionadas à drenagem venosa da glândula tireoide para pronta referência a cirurgiões vasculares e endócrinos. O estudo foi realizado no Departamento de Anatomia, e a pesquisa bibliográfica foi realizada nas bases de dados PubMed, SciELO, ResearchGate, MEDLINE e Scopus. Vários termos relacionados à glândula tireoide e sua drenagem venosa foram considerados para busca na literatura. O levantamento da literatura revelou que as veias tireóideas superior e média apresentam as menores variações em termos de trajeto e terminação, enquanto a veia tireóidea inferior apresenta as maiores variações. O conhecimento detalhado da anatomia normal e de variações anatômicas das veias tireóideas é de extrema utilidade para os cirurgiões vasculares na realização de cirurgia anterolateral do pescoço, especialmente traqueostomia, um procedimento que salva vidas, minimizando complicações intra e pós-operatórias, morbidade e mortalidade.

## INTRODUCTION

The thyroid gland is an endocrine gland located in the lower part of the front and sides of the neck. It regulates basal metabolic rate and stimulates somatic and psychic growth. Thyroid enlargement leading to goiter causes various complications causing patients discomfort and necessitating thyroid surgery. During thyroid surgery, besides surrounding structures, the arteries irrigating the thyroid gland and its venous drainage have to be taken care of to avoid intraoperative and postoperative complications. Besides thyroid surgery,^1^ the veins of the thyroid gland may also be damaged in various other surgical procedures in the neck, like parathyroid and laryngeal surgery, transposition of myocutaneous flaps for reconstruction,^2^ and tracheostomy,^3-5^ with fatal outcomes. This review was planned in consideration of the immense clinical implications associated with damage to the venous drainage pertaining to the thyroid gland. The aim of this review is to conduct a thorough and detailed presentation of normal and variant anatomy of the thyroid veins along with their surgical implications. This information will act as a ready reference and guide for vascular surgeons during surgery involving the anterolateral neck region, thus minimizing iatrogenic injury to the veins of thyroid gland and thereby reducing intraoperative and postoperative complications, fatalities, and mortality rates.

## MATERIAL AND METHODS

The study was conducted at the department of Anatomy, UP University of Medical Sciences, India. The literature was explored using the Pubmed, Scielo, Researchgate, Medline, and Scopus databases. The terms used for literature searches were “Thyroid gland, Anatomy of thyroid gland, Superior thyroid vein, Inferior thyroid vein, Middle thyroid vein, Fourth thyroid vein, Vein of Kocher and venous drainage of thyroid gland, clinical implications of thyroid veins, and significance of venous drainage of thyroid gland”. Standard anatomy text books like Gray’s anatomy and Cunningham’s manual of practical Anatomy were also consulted. After the literature search, all of the information related to venous drainage of thyroid gland was consolidated and interpreted and associated clinical implications were extracted.

### Normal venous drainage of the thyroid gland

**Figure 1:**
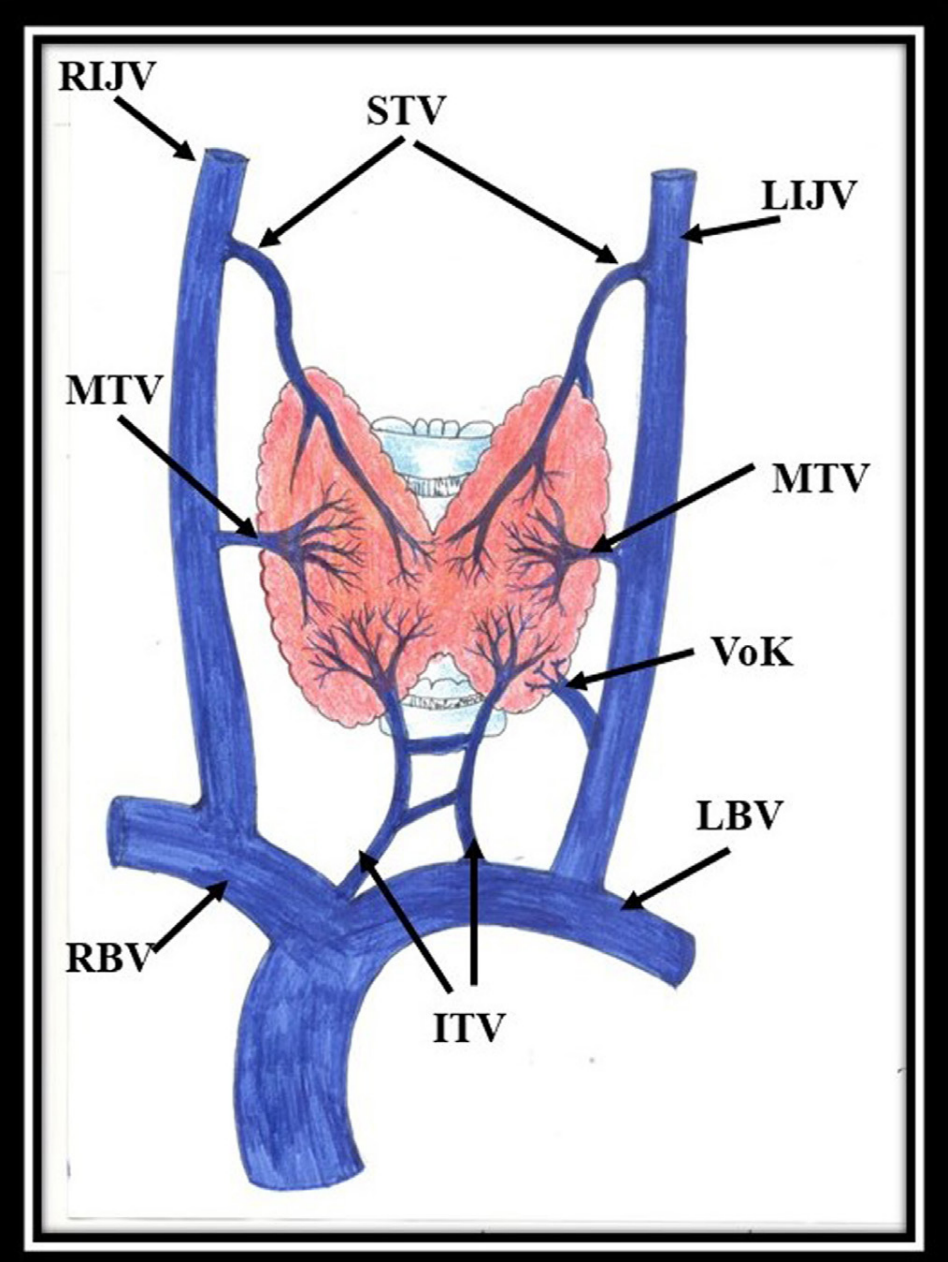
Standard venous drainage of the thyroid gland. RIJV- right internal jugular vein, LIJV- left internal jugular vein, STV- superior thyroid vein, MTV- middle thyroid vein, VoK- vein of Kocher, ITV- inferior thyroid vein, RBV- right brachiocephalic vein, LBV- left brachiocephalic vein.

The thyroid gland is drained by three constant veins, the superior, middle, and inferior thyroid veins, and by a fourth, the thyroid vein of Kocher (Figure 1), which is not always present. Three pairs of thyroid veins originate from the thyroid venous plexus, present deep to the true capsule of thyroid gland. The normal and variant anatomy of the thyroid veins along with their surgical implications are expounded in the following sections.

### Superior thyroid vein anatomy

The superior thyroid vein, also known as the vena thyroidea superior, emerges at the upper pole of the thyroid gland after originating from thyroid venous plexus. The vein passes superiorly and laterally across the superior belly of the omohyoid muscle and the common carotid artery to enter the internal jugular vein alone or in the common facial vein^6^ and is accompanied by the superior thyroid artery. Sometimes, it may also drain into the facial vein. The main tributaries of the superior thyroid vein are the superior laryngeal vein and the cricothyroid vein. The superior thyroid vein mainly collects venous blood from the thyroid gland and the larynx.

### Incidence and variant modes of termination of the superior thyroid vein

The superior thyroid vein is reported to be present bilaterally constantly.^7,8^ More than one superior thyroid vein was observed by Remmert et al.^7^ and was found to be double in 16.7% of cases by Wafae et al.^8^ The superior thyroid vein was found to be formed by primary branches in 80.1% and secondary branches in 19.9% of cases.^8^

The superior thyroid vein was found to drain into the internal jugular vein in 97.2% of cases.^8^ When the vein was single, it opened into the internal jugular vein in 29.4%, while it drained with the lingual vein in 52.1%, with a linguofacial trunk in 35.4%, and with the facial vein 2.1%.^8^ In two cases, the superior thyroid vein was found to end in the vertebral vein.^8^ Chevrel et al.^9^ found the superior thyroid vein ending into the internal jugular vein in 10% of cases and at the linguofacial trunk in 56%, and in both in 33% of cases. The superior thyroid vein was observed to drain into the facial vein in 43% and the rest into the internal jugular vein,^7^ while it was found to drain at the junction of lingual and facial vein in 44%.^10^

Before joining the internal jugular vein, the superior thyroid vein was found to fuse with the lingual vein in 35.4%, with the retromandibular vein in 6.2%, with the retromandibular and facial veins in 4.2%, and with the facial vein in 2.1%.^8^ The superior thyroid vein terminated below the upper margin of the hyoid bone in 85.7%.^8^

### Anatomy, incidence, and variant modes of termination of the middle thyroid vein

The middle thyroid vein is also known as the vena thyreoidea media*.* It is the shortest in length compared to the superior and inferior thyroid veins. The middle thyroid vein arises on the lateral surface of the thyroid gland at the middle of the thyroid lobe. It crosses the common carotid artery and then drains into the internal jugular vein.^6^

This vein may be absent or, very rarely it may be double.^6^ The middle thyroid vein was observed in 29% of cases by Chevrel et al.,^9^ in 43.3% by Wafae et al.,^8^ and in 55.2% by Shima et al.^2^ Mostly, this vein was observed singly bilaterally, but in one case two middle thyroid veins were detected on one side.^8^ The middle thyroid vein was observed in 62% of patients with 80% arising in the middle of the thyroid lobe during thyroid surgery.^11^ Authors found the middle thyroid vein was more frequent in hyperthyroidism and in goiter cases.^11^ Knowledge of the anatomic variability of middle thyroid veins is useful for minimizing the risk of bleeding and preserving laryngeal nerves and parathyroid glands.^11^

As far as termination of the middle thyroid vein is concerned, in most cases, the vein was found to end in the internal jugular vein.^8,12^ While in 1.7% of cases the middle thyroid vein was observed to empty into the vertebral vein.^12^ In most of the cases, the middle thyroid vein was found to pass anterior to the common carotid artery and the recurrent laryngeal nerve, while in one case it was detected lying posterior to the recurrent laryngeal nerve.^8^

### Anatomy, incidence, and variant modes of termination of the inferior thyroid vein

The inferior thyroid vein emerges at the lower border of the isthmus of the thyroid gland. Inferior thyroid veins were observed to occur in pairs; right and left, descending down, anastomosing with each other, and forming a pre-tracheal venous plexus or plexus thyroidea impar anterior to the trachea and deep to the sternothyroid muscle.

**Figure 2:**
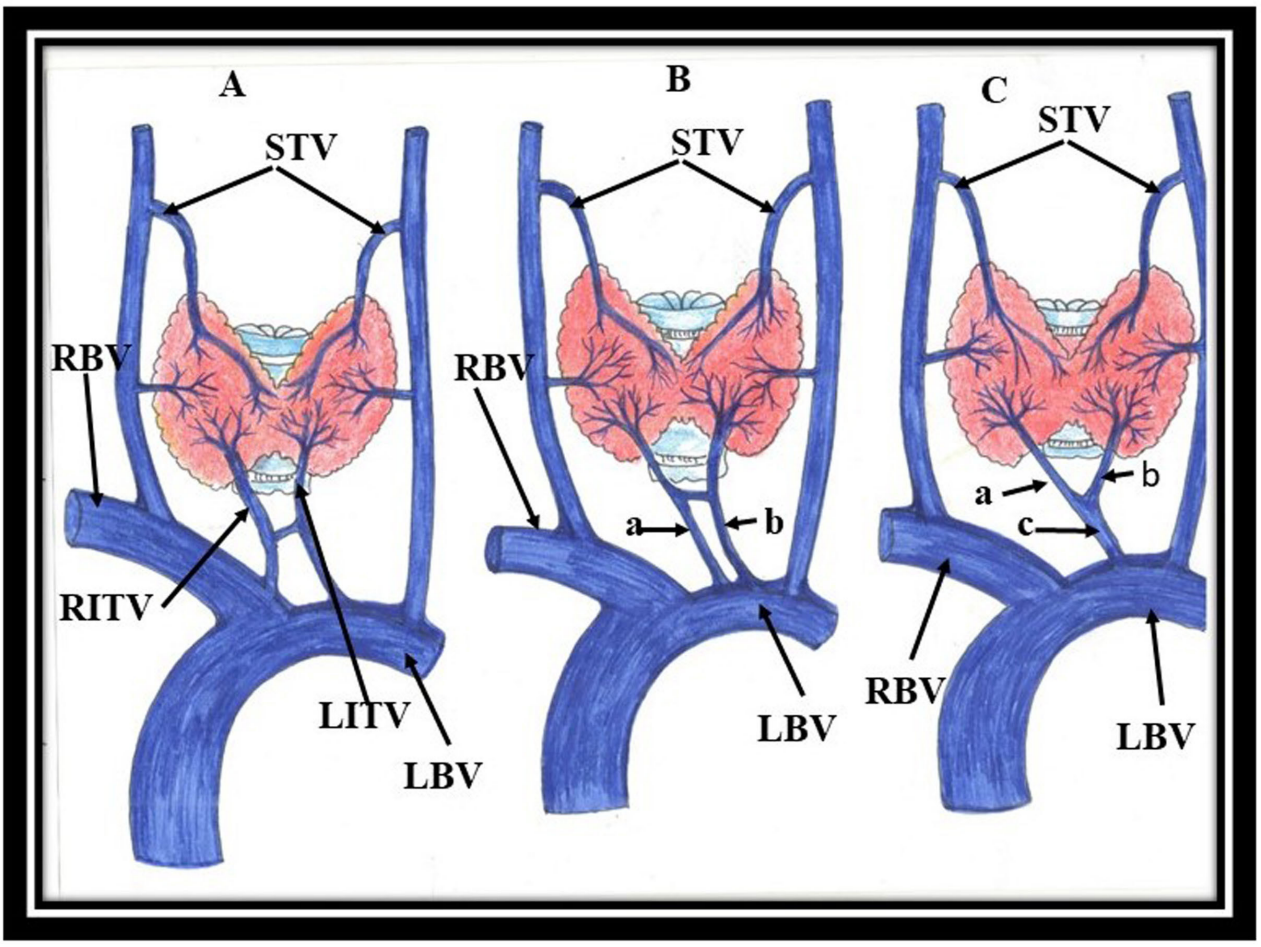
Variant terminations of inferior thyroid veins. **(A)** Standard termination of the inferior thyroid vein; **(B)** Right inferior thyroid vein (a) and left inferior thyroid veins (b) opening into left brachiocephalic vein; **(C)** Right (a) and left (b) inferior thyroid veins fuse forming inferior thyroidea ima vein (c) terminating into left brachiocephalic vein.

The right and left inferior thyroid veins are the largest of the three thyroid veins and are found to be asymmetric and present the most variant patterns in terms of number, course, and termination.^6^ They follow different paths on each side as illustrated below (Figure 2).

After originating from the pre-tracheal plexus, the right inferior thyroid vein travels anterior to the innominate artery (right brachiocephalic artery) and posterior to the sternothyroid muscle, draining into the right brachiocephalic vein (Figure 2A). Infrequently, it passes in front of the trachea and drains into the left brachiocephalic vein (Figure 2B). The left vein crosses the trachea to enter the left brachiocephalic vein. Rarely, both inferior thyroid veins form a common trunk called the thyroid ima vein, emptying into the left brachiocephalic vein^6^ (Figure 2C). Many investigators found that the inferior thyroid vein occurred constantly in all specimens observed,^1,4,13^ while it was observed in 90% of cases with computed tomography,^14^ and in 96.7% of cadavers.^8^ Besides variant course, variations in the number of inferior thyroid veins have also been reported and are tabulated in Table 1. In addition to variations in number, large variability is also observed in the termination of inferior thyroid vein, as shown in Table 2.

**Table 1:** Numbers and percentages of inferior thyroid veins as reported by various authors.

**S. No**	**Number of ITV**	**Percentage**	**Author**
1.	1	10	Krausen^4^
	2	10	
	3	40	
	4	20	
	5	20	
2.	1	81.5	Lázaro da Silva et al.^1^
	2	18.5	
3.	1	38	Belli et al.^14^
	2	33	
	3	16	
	4	3	
4.	1	60.7	Moriggl and Pomaroli^13^
	2	30.3	
	3	8.4	
	4	0.6	
5.	1	62.1	Wafae et al.^8^
	2	27.6	
	3	3.4	
	4	3.4	
	5	3.4	

ITV- inferior thyroid vein. S. No- serial number.

**Table 2:** Variant terminations of the inferior thyroid vein as reported by various authors.

**S. No.**	**Author**	**Mode of termination of inferior thyroid vein**						
		Right BV	Left BV	Junction	Right and left BV	Right BV and junction	Left BV and junction	Right, left BV and junction
1.	Moriggl and Pomaroli^13^	6%	47%	20.2%	10.7%	1.8%	9.5%	3%
2.	Krausen^4^	1/10	7/10	NA	NA	NA	NA	NA
3.	Chevrel et al.^9^	+	+	NA	NA	NA	NA	
4.	Lázaro da Silva et al.^1^	+	+	+	NA	NA	NA	NA
5.	Wafae et al.^8^	26.1%	60.9%	NA	13.0%	NA	NA	NA

BV-Brachiocephalic vein, junction- junction between two brachiocephalic veins; NA= Not available.

Wafae et al.^8^ related the inferior thyroid vein to the tracheal rings and found that 80.4% of these veins were located between the 7th and 12th rings.

Based on the termination of the inferior thyroid vein, Moriggl and Pomaroli^13^ classified inferior thyroid veins into three categories, as detailed below:

1. Draining exclusively into the brachiocephalic vein (Right, left, and junction);
2. Right and left, left and junction, right and junction, right, left and junction;
3. Drainage into other veins of the mediastinum.

Table 2 lists incidence rates of various types of inferior thyroid vein termination as reported by various authors.

Belli et al.^14^ described termination of inferior thyroid veins in two classes:

1. Veins flow in the lower part of the neck and form two veins that unite and end in the proximal part of the brachiocephalic vein (60%);
2. The inferior thyroid veins do not unite and end respectively in the left brachiocephalic vein and in the junction of the right brachiocephalic vein with the superior vena cava.

Rarely, the inferior thyroid vein may also drain into the superior vena cava

The tributaries of the inferior thyroid veins are the esophageal, tracheal, and inferior laryngeal veins, which contain valves at their opening into the brachiocephalic veins. The inferior thyroid veins drain the cervical part of the esophagus, the distal part of the larynx, and the proximal part of the trachea.

### Fourth thyroid vein of Kocher

Sometimes, besides the three thyroid veins mentioned in preceding sections, a fourth thyroid vein of Kocher is observed to emerge between middle and inferior thyroid veins draining into the internal jugular vein (Figure 1). This vein is scantly reported.

### Significance of variant anatomy of thyroid veins

Detailed and exhaustive knowledge of the thyroid veins is important during parathyroid, cricothyroid, and laryngeal surgeries and in transposition of myocutaneous flaps for reconstructions^2^ and in tracheostomies,^3-5^ since these veins are vulnerable during the aforementioned surgical interventions.

Increased incidence of massive hemorrhage is observed during tracheostomy procedures both in emergency and routine tracheostomies due to iatrogenic injury to the inferior thyroid vein.^4^ A case of fatal hemorrhage was reported caused by injury to the inferior thyroid vein during percutaneous tracheostomy.^3^ Tracheostomy failure resulting in massive hemorrhages due to injury to vessels of the thyroid gland was observed in 4.8% of cases, which is quite high.^5^ Ultrasonography is therefore recommended for evaluation of variant thyroid vein anatomy before proceeding with a tracheostomy procedure.^5^ There is a paucity of literature describing detailed anatomical variations of the thyroid veins, so before proceeding with neck surgeries, especially thyroid and laryngeal surgeries and tracheostomies, diagnostic imaging should be conducted in the form of MRI and ultrasound^14,15^ to avoid complications both during and after surgery, since when percutaneous dilatational tracheostomy was performed with color Doppler ultrasound, reduced intraoperative blood loss was observed (not exceeding 8 ml), thus improving success rate and reducing mortality.^16^ The middle thyroid vein crosses the common carotid artery and recurrent laryngeal nerve. This fact should be kept in mind during thyroid surgery to avoid injury to these related structures. In addition to this, a middle thyroid vein was more frequently observed in hyperthyroidism and goiter cases.^11^ Hence, the authors of this study speculate that there is a relationship between presence of a middle thyroid vein and hyperthyroidism and goiter, which should be confirmed by further studies.

In view of the immense clinical implications of variant anatomy of the thyroid veins, further studies are recommended to decrease mortality due to lack of information on thyroid veins.

## CONCLUSION

The superior thyroid vein is found to present constantly and is normally single on both sides and opens into the internal jugular vein along with other tributaries of the internal jugular vein. Very few variations of this vein in terms of course and termination are described in literature. The middle thyroid vein may be absent in some cases, but when present crosses the recurrent laryngeal nerve and the common carotid artery. This fact should be kept in mind during thyroid and laryngeal surgeries to avoid inadvertent injury to these structures. The inferior thyroid vein is found constantly, but it exhibits the greatest range of variations in terms of number and mode of termination. Besides this, the inferior thyroid vein is related to the anterior surface of trachea. This fact demands attention of surgeons to avoid lesions to this vein in tracheostomies.

Thus, to avoid injury to thyroid veins during surgical procedures of the neck region, particularly thyroid and laryngeal surgeries and tracheostomies, radiological imaging and especially color Doppler ultrasound are recommended before proceeding with surgery in these regions to avoid fatal hemorrhage and reduce mortality, since the literature suggests that when tracheostomies are performed under color Doppler ultrasound guidance there is reduced blood loss during surgery, thus decreasing mortality and improving success rates.
